# Longer Years of Practice and Higher Education Levels Promote Infection Control in Iranian Dental Practitioners

**Published:** 2012-07-30

**Authors:** M Ebrahimi, B M Ajami, A Rezaeian

**Affiliations:** 1 Department of Pediatric Dentistry, Dental Research Center, Dental School, Mashhad University of Medical Sciences, Mashhad, Iran; 2 Dentist, Mashhad, Iran

**Keywords:** Dentistry, Education, Infection control

## Abstract

**Backgrounds:**

Infection control is one of the primary responsibilities of dental health care personnel. The purpose of this study was to evaluate whether the infection control practices of Iranian dentists and dental nurses working in governmental dental health care centers were influenced by their educational level and years of practice.

**Methods:**

This cross-sectional analytical study was completed in 2009, and it included 63 Iranian dental practitioners. Infection control knowledge was evaluated with a self-administered questionnaire, and infection control practices were evaluated with a checklist of questions by observation with one researcher.

**Results:**

The dental practitioners in Mashad had a low level of infection control knowledge. Dental personnel with a higher educational level had significantly greater knowledge than those with less education. Additionally, dental personnel who had more years of practice had a greater knowledge of infection control.

**Conclusion:**

Since dental practitioners working in Mashad governmental dental health care centers with fewer years of practice and less educational level had a low level of infection control knowledge, we recommend a continuing educational program for this group and dental nurses.

## Introduction

Infection control is one of the primary responsibilities of dental health care personnel (DHCP). The mouth’s natural flora consists of a vast number of microorganisms. Since dental procedures result in the spread of blood and saliva, infection control is an essential practice in dentistry.[1] There is a growing concern that bacterial aerosols spread through the entire dental room during dental procedures.[2]

Due to these concerns, dental practitioners should wear personal protective equipment, such as gloves, facial masks and eye protection during daily practice to reduce their exposure to blood born pathogens.[3] Using gloves is one of the most effective protective measures for reducing disease transmission in dentistry.[4] Previous studies have reported that between 56% and 100% of dental practitioners wore gloves.[5][6][7][8][9][10][11][12] Additionally, wearing a disposable facial mask is generally recommended for all procedures producing aerosols and to provide a safe environment for practitioners.[4] Several previous studies reported that the percentage of dental practitioners using face masks ranged from 32% to 90.1%.[5][6][7][9][11][12][13][14] Wearing eye protection is another important consideration, since it protects the operator from aerosols, debris and potentially infective particles.4 In previous reports, the number of practitioners who wore protective eye wear ranged from 14.7% to 91.2%.[6][7][9][11][13][14][15]

Generally, standard precautions have not been used in many countries.[3][4][6][8][11][16][17][18][19][20] Low compliance with current infection control recommendations has been demonstrated in several studies.[21][22][23][24] It was emphasized that in spite of the highest rate of infectious disease in South Africa, the level of knowledge and practice in regard to infection control among dental practitioners was low.[24] However, recently there has been an increasing compliance for infection control in dental procedures. A report by Scully showed that dental practitioners had a high level of knowledge concerning infection control.[8] Also a survey in US Air Force, dental clinics concluded high compliance towards infection control guidelines.[25] Meanwhile, Fabiani reported that dental practitioners needed continuing education to prevent cross-infections.[26] A recent report showed improved compliance with recommended infection control policies in Jordanian dentists.[11] Another recent report showed that acceptance of infection control guidelines has increased in orthodontists in comparison to previous studies, but compliance remained less than ideal.[6] Additionally, it was reported that orthodontists in UK effectively followed infection control guidelines.[27]

It was shown that successful infection control in dental practice primarily depends on dental assistants.[28] Dental nurses have been reported to have a low level of knowledge of infection control in researches.[10][29] Several studies showed that Iranian dental practitioners had poor attitudes and practices towards infection control.[16][17][18][30] To date, however, there have been a limited number of studies on infection control practices in Iranian dental settings. A large segment of the population use governmental dental care centers because of their affordability. Therefore, in this study, we aimed to evaluate the infection control practices and knowledge of Iranian dental practitioners working in governmental dental health care centers.

## Materials and Methods

This cross-sectional, analytical study included all of dental practitioners (N=63) working in 38 governmental dental health care centers in Mashhad, Iran in 2009. There were 38 dental health care centers in different areas of Mashhad city. The sampling method was the convenience method. The inclusion criteria included dental practitioners working in governmental health centers in Mashhad, Iran. The dental practitioner’s workings in private offices or clinics in Mashhad and those who lacked desire to take part in this research were excluded from the study. The questions aimed to evaluate two aspects: Knowledge (33 questions) and practices (36 questions). Knowledge was evaluated using questionnaires that were filled by dental practitioners, and practices were evaluated by a researcher using a checklist of questions with observation (Table 1). A pilot study was performed on 10 randomly selected dental practitioners from the study group. The correlation coefficient was 82%, and test-retest reliability was high. The questionnaire had an acceptable validity according to the pediatric dentistry faculty. The questions measured the knowledge and practices of the dental practitioners. One point was given for each correct answer, one point was subtracted for each incorrect answer, and zero points were given for questions with no answer. Subsequently, a mean score (between 0 and 1) was calculated for each dental practitioner within the two tested groups. Knowledge was classified in five levels: Very low (0-0.2), low (0.2-0.4), intermediate (0.4-0.6), high (0.6-0.8) and very high (0.8-1). In addition, practices were scored as weak (0-0.33), average (0.33-0.66) and good (0.66-1).

**Table 1:** Questionnaires in the study group.

**Part 1: **Knowledge evaluation in the study group
1) Which groups should be immunized against Hepatitis B?
a) Society, b) People who deal with blood, c) Dentists, d) Laboratory and hospital staffs,
2) How do you know that a person with history of Hepatitis B has been recovered?
a) He is asymptom, b) 6 months after disease has been passed, c) 1 year after disease has been passed, d) HBS Ag-, Anti HBS+
3) Before Immunization of HBV, measuring HBS Ag is recommended for:
a) Prevalence is more than 60% , b) Prevalence is more than 30%, c) Not recommended, d) High risk people
4) What is the most common clinical symptom of chronic Hepatitis C?
a) Fatigue, b) Jaundice, c) Itching, d) Abdominal pain.
5) Which of the following does not exacerbate HCV infection?
a) Simultanous HCV and HAV, b) Simultanous HCV and HBV, c) Alcohol, d) Aging.
6) Which of the following is at the highest risk for a dentist? a) Tuberculosis, b) HIV, c) Hepatitis B, d) a and b.
7) Prevention from which is simpler? a) Hepatitis B, b) HIV, c) Both of them, d) No matter.
8) What is the protection of society against HIV virus?
a) Immunization, b) No treatment of HIV+ patients, c) Following infection control guidelines, d) No using syringe and local anesthesia.
*) Which of the following methods could be used for disinfection/sterilization?
I) Water and soap, II) Alcohol, III) Microten, IV) Deconex, V) Autoclave, VI) Oven VII) Glutaraldehyde (as disinfectant agent), VIII) glutaraldehyde (as sterilizing agent).
*(each question may have more than one answer).
9) Instruments, 10) High speed turbine, 11) Low speed turbine, 12) Injection syringe,
13) Handpieces, 14) Amalgam carrier, 15) Burs, 16) Files and brooches, 17) Lentulo spiral, 18) Matrix holder, 19) Eye protection, 20) Mouth probe, 21) Clamp, 22) Punch and forceps, 23) Elevator and forceps, 24) Light cure system, 25) Amalgamator, 26) Radiographic films, 27) Shields, 28) Acrylic tray, 29) Metal tray.
*) Which of the recommended disinfectants could be used for the following?
I) Quaternary ammonium compounds, II) Alcohol, III) Iodophor solution, IV) Hypochlorite sodium, V) Glutaraldehyde (as disinfectant agent), VI) Fenoles, VII) Deconex, VIII) Microten.
30) Different parts of dental unit, 31) Dental unit lamp handle, 32) Dental chair, 33) Floor.
**Part 2: Evaluation of behavior:**
1- Dental practitioners wear gloves during dental procedures? a) Yes, b) No
2- Dental practitioners wear mask during dental procedures? a) Yes, b) No
3- Dental practitioners wear protective eye during dental procedures? a) Yes, b) No
4- Dental practitioners wear shield during dental procedures? a) Yes, b) No
5- Personnel's gowns are clean and appropriate: a) Yes, b) No
6- Gown for patients: a) is not used, b) is not disinfected, c) is disposable, d) is disinfected
7- Personal health is appropriate: a) Yes, b) No
8- Dental clinic is ventilated: a) Yes, b) No
9- There is enough mask in dental clinic: a) Yes, b) No
10- There are enough gloves in dental clinic: a) Yes, b) No
11- There are enough shields in dental clinic: a) Yes, b) No
12- There is enough protective eye in dental clinic: a) Yes, b) No
13- Collecting of garbage is done correctly: a) Yes, b) No
14- There are insects and beetles in dental clinic: a) Yes, b) No
15- Cleaning status is satisfactory in dental clinic: a) Yes, b) No
16- Lavatory is satisfactory in clinic: a) Yes, b) No
17- Pantry is satisfactory in clinic: a) Yes, b) No
18- Disinfectants have production and expiry date: a) Yes, b) No
19- There are safety boxes in dental clinic: a) Yes, b) No
20- Personal instruments are used for each patient: a) Yes, b) No
21- Disposable unit covers are used for each patient: a) Yes, b) No
22- Hands are washed after removing gloves at the end of dental treatment for each patient? a) Yes, b) No
23- Sterilizable high and low speed turbines are used for each patient: a) Yes, b) No
24- There is washing liquid in the lavatory: a) Yes, b) No
25- There is a special room for washing of infected items: a) Yes, b) No
26- Disinfectant solutions are labeled: a) Yes, b) No
27- There are guidelines for dilution of disinfectants: a) Yes, b) No
28- There is a special sanitary room for disinfectant solutions: a) Yes, b) No
29- Safety boxes have biohazard sign: a) Yes, b) No
30- Infected patients (such as HBS Ag+) are treated in an isolated room: a) Yes, b) No
31- After injection, needles are removed in a safety box: a) Yes, b) No
32- Waste container is washable: a) Yes, b) No
33- There is gated trash in the ward: a) Yes, b) No
34- Which of the following items are used as disinfectant in dental clinic?
I) Quaternary ammonium compounds, II) Alcohol, III) Iodophor solution, IV) Hypochlorite sodium, V) Glutaraldehyde (as disinfectant agent), VI) Fenoles, VII) Deconex, VIII) Microten
35- Autoclave is used for sterilization? a) Yes, b) No
36- Oven is used for sterilization? a) Yes, b) No

The statistical package of SPSS (Version 11.5, Chicago, IL, USA) was used for data analysis. Data were collected and analyzed in tables and charts using tendency parameters and dispersion measures, including the t-test, one-way analysis of variance (ANOVA) and the Chi-Square test. Statistical significance was based on probability p values of ≤ 0.05. T-test was used for comparison of knowledge and practice scores between dental practitioners according to two educational levels. The ANOVA was used for comparing knowledge and practice scores between dental practitioners with different years of practice. Chi-Square test was used to compare wearing protective barriers in dental practitioners.

## Results

The members of the study group were categorized according to their educational levels into two subgroups: Doctorate (n=41) and diploma (n=22). There were a few dental nurses with bachelor’s (n=1) and associate degrees (n=2), who were placed in the diploma degree subgroup (n=18). The educational level of one practitioner was not specified. Our findings regarding the knowledge and practices of Iranian dental practitioners were categorized in the two following sections.

**Fig. 1:**
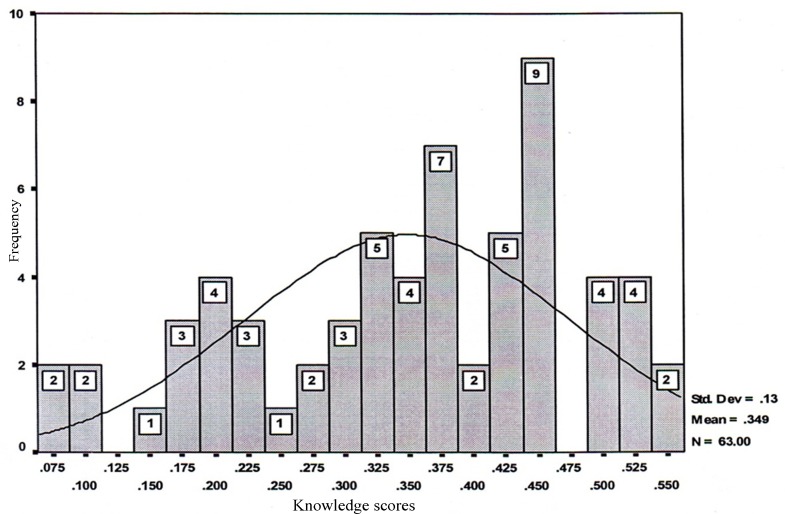
Dental practitioners' infection control knowledge scores.

**Fig. 2:**
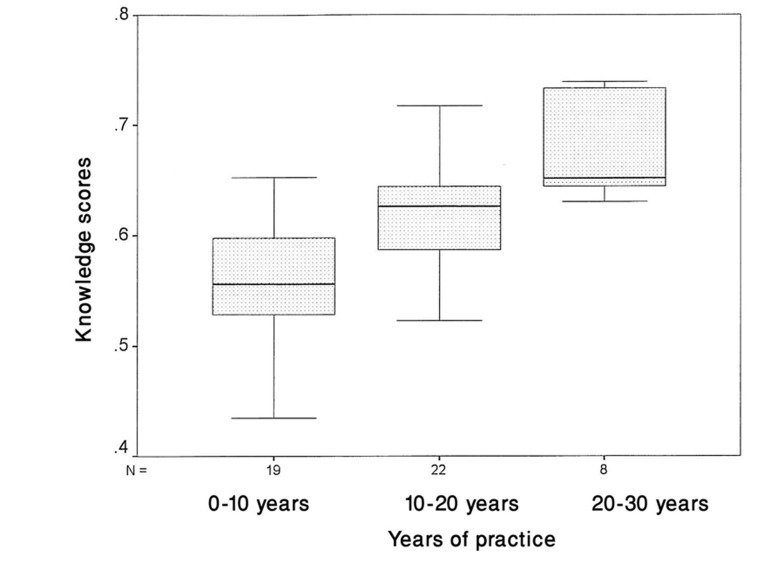
Dental practitioners' infection control knowledge scores in relation to their years of practice.

Infection control knowledge: The response rate for the questionnaire was 100%. Figure 1 shows a histogram of the knowledge scores in our study group. The Iranian dental practitioners had a mean knowledge score of 0.34±0.13, indicating a low overall knowledge of infection control. Dental nurses with diploma degrees had a low knowledge (0.27±0.20), and dentists with doctorate degrees had an intermediate knowledge (0.40±0.15) of infection control. The dental staff with doctorate degrees had a significantly higher level of knowledge than the staff with diploma degrees (p=0.028). As shown in Figure 2, the mean levels of knowledge in DHCP with less than 10 years experience, 10-20 years, and more than 20 years were 0.31±0.14, 0.37±0.16, and 0.40±0.13, respectively. One-way ANOVA showed that the group with more than 20 years of experience had a higher level of knowledge concerning infection control than the other two other groups (p=00.1)

**Fig. 3:**
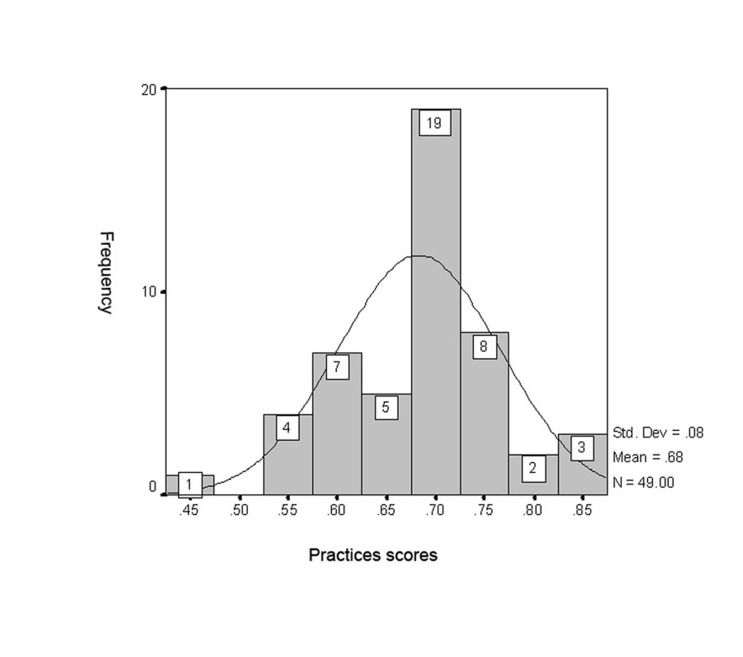
Dental practitioners' infection control practices scores

Infection control practices: In our evaluation of the practices of DHCP, 77.7% (49 out of 63) of the dental practitioners were willing to participate. As shown in Figure 3, the mean score for personnel practices was 0.68±0.83, indicating that the infection control practices were good. Dentists had a mean practice score of good (0.72±0.62), and dental nurses had a mean score in the average range (0.64±0.08). Personnel with doctorate degrees had better infection control practices than the personnel with diplomas (t-test, p=0.000).

**Fig. 4:**
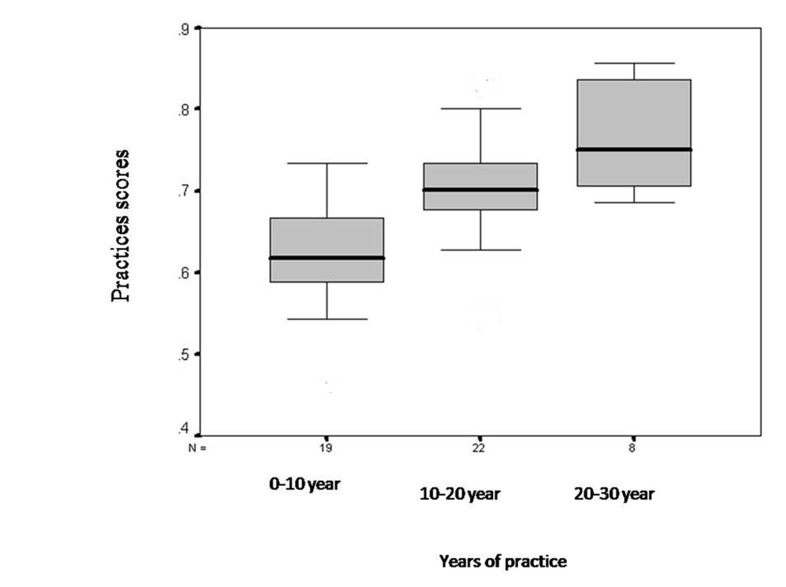
Dental practitioners' infection control practices scores in relation to their years of practice.

Figure 4 shows a comparison of the dental practitioners’ practices scores in three groups with different years of experience. The mean practices scores in personnel with less than 10 years experience, 10-20 years of experience and more than 20 years of expe-rience were 0.63±0.06, 0.7188 ±0.63 and 0.76±0.07, respectively. One-way ANOVA showed that the groups with 10-20 years experience and more than 20 years of experience had better infection control practices than the other group.

Table 2 shows the usage of protective barriers by dental practitioners. We observed no significant differences in the use of facial masks and gloves in practitioners with different educational levels. However, we found that dentists used protective eyewear and shields significantly more often than dental nurses (p=0.000).

**Table 2:** Rates of DHCPs wearing protective barriers.

**Protective barrier**		**Gloves**		**Masks**		**Eye protection**		**Shield**	
**Degree**		**-**	**+**	**-**	**+**	**-**	**+**	**-**	**+**
Diploma	No.	0	21	0	21	17	4	19	2
	%	0	100	0	100	81	19	90.5	9.5
Doctorate	n	1	27	1	27	0	28	6	22
	%	3.6	96.4	3.6	96.4	0	100	21.4	78.6
Total	No.	1	48	1	48	17	32	25	24
	%	2.05	97.95	2.05	97.95	34.70	65.30	51.03	48.97

## Discussion

In many countries, infection control practices are inadequate, and there is a limited understanding of the current recommendations.[3][4][6][16][17][18][19][20][21][22][23][24][31] In addition, it was reported that there was low compliance with infection control guidelines in Asian dentists.[31] In our study, we observed that Iranian dental practitioners working in governmental dental care centers in Mashhad city had a poor knowledge of infection control, which was comparable to previous studies in Iran.[16][17][18][30] Therefore, increasing their knowledge regarding infection control seems necessary.

Our study demonstrated that the mean knowledge score of personnel with doctorate degrees was significantly higher than in personnel with diploma degree. Since dental nurses have been shown to have a vital role in infection control, the proper instruction of all dental staff is necessary.[28][32] We observed that the mean practice score of the Iranian dental staff was in the good range, which was higher than in previous studies.[16][17][18] Considering the more importance of infection control in dental procedure, and also regular obligatory courses towards infection control might be the major reasons for promotion in recent years. Practitioners with doctorate degree had significantly higher practice scores than staffs with diploma degree, suggesting that since dentists have a higher knowledge, this results in better practices.

Our findings showed a discrepancy between knowledge and practice in dental governmental centers, which is comparable to a previous study.[33] In our study, the practice score was better than the knowledge score, which may be due to a number of factors. One possibility is the discrepancy in results from compulsory rules in dental governmental sectors that necessitates the use of gloves and masks. Previous reports showed that clinicians with knowledge of infection control and suitable facilities still used poor cross-infection control measures.[4][17]

In spite of the concerns about infectious diseases, standard precautions were not carried out routinely in some dental practices in Iran.[18] The vast majority of DHCP in our study used gloves and facial masks, and these rates were higher than in previous studies in Iran.[18] However, there was a low compliance with wearing eye protection in our study, which is comparable to a previous report.[6] Our rate of eye protection use was lower than a number of studies where the rates ranged from 68% to 91.2%.[7][9][15] Additionally, the American Dental Association has recommended that dental practitioners wear eye glasses with a lateral shield.[34] Therefore, further efforts are necessary to promote the use of protective eyewear by Iranian dental practitioners. We recommend that infection control principles should be taught early in dental school training.

Our study demonstrates that infection control knowledge and practices are significantly higher in practitioners with more years of experience. Therefore, to promote standard infection control practices, it is necessary to establish infection control programs that target newly graduated dental practitioners. In spite of the low knowledge in the study group, the fact that they used some protective barriers was encouraging, and we should consider several related points. First, universal precautions, such as facial masks and gloves, should be provided by employers. Additionally, since only 77.7% of practitioners agreed to participate in our study and since our results were gathered from dentists and dental nurses working in the governmental sector, these results are not representative of all the DHCP in Iran. In spite of the concerns regarding infection transmission, our results indicate that compliance with infection control policies during dental procedures is low in Iran.

Recently, a systematic review showed a positive trend towards infection control compliance in some dental fields.[32] Moreover, several studies showed high compliance with infection control guidelines in dental practitioners.[25][26][27] Considering these steady increases in infection control, we believe the current situation is improving. Our results demonstrated that Iranian DHCP working in governmental dental health care centers in present study had a low knowledge of infection control, but used good infection control practices. Educational programs and regulatory efforts are necessary to promote infection control policies. We especially recommend education on infection control for dental practitioners with fewer years of practice and lower educational levels. Lastly, we recommend systematic personnel assessments on infection control knowledge and practices.
